# Transglucosidase improves the gut microbiota profile of type 2 diabetes mellitus patients: a randomized double-blind, placebo-controlled study

**DOI:** 10.1186/1471-230X-13-81

**Published:** 2013-05-08

**Authors:** Makoto Sasaki, Naotaka Ogasawara, Yasushi Funaki, Mari Mizuno, Akihito Iida, Chiho Goto, Satoshi Koikeda, Kunio Kasugai, Takashi Joh

**Affiliations:** 1 Department of Gastroenterology, Aichi Medical University School of Medicine, 1-1 Yazakokarimata, Nagakute, Aichi 480-1195, Japan; 2 Department of Health and Nutrition, Faculty of Health and Human Life, Nagoya Bunri University, Nagoya, Japan; 3 Department of Frontier Research, Amano Enzyme Inc, Kakamigahara, Japan; 4 Department of Gastroenterology and Metabolism, Nagoya City University Graduate School of Medical Sciences, Nagoya, Japan

**Keywords:** Hemoglobin A1c, Transglucosidase, Type 2 diabetes mellitus, Intestinal microbiota

## Abstract

**Background:**

Recently, the relationship between gut microbiota and obesity has been highlighted. The present randomized, double-blind, placebo-controlled study aimed to evaluate the efficacy of transglucosidase (TGD) in modulating blood glucose levels and body weight gain in patients with type 2 diabetes mellitus (T2DM) and to clarify the underlying mechanism by analyzing the gut microbiota of T2DM patients.

**Methods:**

This study included 60 patients who received placebo or TGD orally (300 or 900 mg/day) for 12 weeks, and blood and fecal samples were collected before and after 12 weeks. Comparisons of fecal bacterial communities were performed before and after the TGD treatment and were performed between T2DM patients and 10 healthy individuals, using the terminal-restriction fragment length polymorphism analysis.

**Results:**

The *Clostridium* cluster IV and subcluster XIVa components were significantly decreased, whereas the *Lactobacillales* and *Bifidobacterium* populations significantly increased in the T2DM patients compared with the healthy individuals. By dendrogram analysis, most of the healthy individuals (6/10) and T2DM patients (45/60) were classified into cluster I, indicating no significant difference in fecal bacterial communities between the healthy individuals and the T2DM patients. In the placebo and TGD groups, the bacterial communities were generally similar before and after the treatment. However, after 12 weeks of TGD therapy, the Bacteroidetes-to-Firmicutes ratio in the TGD groups significantly increased and was significantly higher compared with that in the placebo group, indicating that TGD improved the growth of the fecal bacterial communities in the T2DM patients.

**Conclusions:**

Therefore, TGD treatment decreased blood glucose levels and prevented body weight gain in the T2DM patients by inducing the production of oligosaccharides in the alimentary tract and modulating gut microbiota composition.

**Trial registration:**

UMIN-CTR UMIN000010318

## Background

Previously, we introduced a novel strategy using *Aspergillus niger* transglucosidase (TGD) to produce oligosaccharides from starch in the digestive tract of humans to decrease postprandial blood glucose levels in individuals with impaired glucose tolerance and at high risk of developing type 2 diabetes mellitus (T2DM) [1]. Furthermore, we demonstrated that TGD administration decreases glycosylated hemoglobin (HbA1c) and insulin levels in T2DM patients [2]. We suggested that the mechanism underlying the reduction in the total amount of orally ingested calories is the consequent transformation of digestible substrate to indigestible fiber in the alimentary tract. Our human microbial genomes encode many metabolic capacities that we have not fully evolved. There may be evidence to clarify why unabsorbable carbohydrates improve postprandial hyperglycemia in diabetes patients [3], which is also the mechanism that explains the effects of TGD. Recently, there is increasing evidence that is indicative of the relationship between obesity and gut microbiota [4-7]. Comparisons of the gut microbiota between genetically obese mice and their lean littermates, and between obese and lean human volunteers revealed that obesity is associated with changes in the relative abundance of the 2 dominant bacterial divisions, Bacteroidetes and Firmicutes. Colonization of adult germ-free mice with a distal gut microbial community harvested from conventionally raised mice produced a dramatic increase in body fat within 10–14 days, despite an associated decrease in food consumption [4]. These findings have led us to propose that the gut microbiota of obese individuals may be more efficient at extracting energy from a given diet than those of lean individuals.

This study aimed to assess the gut microbiota of T2DM patients and healthy individuals by terminal-restriction fragment length polymorphism (T-RFLP) analysis of fecal samples. Furthermore, we aimed to clarify the mechanism of the effect of TGD treatment by analyzing fecal microbiota and comparing fecal microbiota composition before and after TGD treatment.

## Methods

The present study was designed as a randomized, double-blind, placebo-controlled trial and was conducted in Japan. The ethics review committee of the Nagoya City University Graduate School of Medical Sciences granted approval of this study, and informed consent was obtained from all the subjects. TGD (3,000,000 U/g) was purchased from Amano Enzyme Inc. (Nagoya, Japan). Two types of capsules containing TGD (50 and 150 mg) and a placebo capsule were prepared. Patients took 2 capsules after every meal for 12 weeks, and fecal and blood sampling was performed before and at the end of the study. The patient enrollment criteria and study design used were described previously [1]. Briefly, all the eligible patients had an established diagnosis of T2DM according to the Japanese Diabetes Society’s criteria for diabetes mellitus; HbA1c levels of 5.8–7.5% at screening; stable dosages of medication for at least 1 month; and stable diabetes condition for at least 3 months (change in HbA1c level by <1.0%). We excluded patients who had a history of gut resection. Based on our previous in vitro and in vivo experiments [2,8], we used TGD dosages of 300 and 900 mg/day. Using simple randomization technique, the patients were randomized into 3 groups (1:1:1 proportion) according to the treatment received as follows: 100 mg of TGD, 300 mg of TGD, and placebo 3 times a day after the main meal for 12 weeks. The capsules containing transglucosidase and placebo were prepared by Adaptogen pharmaceutical Co., LTD (Tajimi, Japan), and allocation was blinded until the end of study. The statistician generated the randomization list managed by clinical research coordinator. Blood and feces samplings were performed before and at the end of the study. The primary outcome was the change in HbA1c level (previously reported). Patients whose fecal samples were collected before the treatment in our previous study were included in the evaluation. Important secondary outcomes included the change in various other metabolic parameters and fecal microbiota. For comparison, fecal samples were collected from healthy volunteers, who had no abnormality as determined by medical examination. Fecal microbiota were analyzed using T-RFLP. To evaluate nutrient intake, a data-based short food frequency questionnaire [9] was used. The patients received stable dosages of diabetes medication throughout the study. The sample size calculation was based on a previous study [2,6], and based on alpha of 0.05 with a power of 80%. Taking into account a drop-out of 10%, a total sample size of 66 patients will be randomized.

### Fecal DNA extraction

The fecal samples were suspended in 4 M guanidinium thiocyanate, 100 mM Tris–HCl (pH 9.0), and 40 mM EDTA after washing 3 times with sterile distilled water and then beaten with glass beads, using a mini bead beater (BioSpec Products). Thereafter, DNA was extracted from the bead-treated suspension using benzyl chloride, as described by Zhu et al. [10]. The DNA extract was then purified using a GFX polymerase chain reaction (PCR) DNA and Gel Band Purification Kit (Amersham Biosciences). The final concentration of each DNA sample was adjusted to 10 ng/μL.

### T-RFLP analysis

The amplification of the 16SrDNA, digestion of restriction enzymes, size fractionation of T-RFs, and analysis of T-RFLP data were performed according to the protocol described by Nagashima et al. [11]. Briefly, polymerase chain reaction (PCR) was performed using the total fecal DNA (10 ng/μL) and primers of 516f (5′-TGC CAGCAGCCGCGGTA-3′; *Escherichia Coli* positions, 516–532) and 1510r (5′-GGTTACCTTGTTACGACTT-3′; *E. coli* positions, 1510–1492). The 5′-ends of the forward primers were labeled with 6′-carboxyfluorescein, which was synthesized by Applied Biosystems (Tokyo, Japan). The amplified 16S rDNA genes were purified using the GFX PCR DNA and Gel Band Purification Kit (GE Healthcare Bio-Sciences, Tokyo, Japan) and redissolved in 30 μL of distilled water. The purified PCR products (2 μL) were digested with 10 U of *Bsl*I at 55°C for 3 h. The length of the T-RF was determined using an ABI PRISM 3130 × 1 genetic analyzer (Applied Biosystems, CA, USA) in GeneScan mode. Standard size markers were used (MapMarker X-Rhodamine Labeled 50–1000 bp, BioVentures, TN, USA). The fragment sizes were estimated using the local southern method of the GeneMapper software (Applied Biosystems, CA, USA). The T-RFs were divided into 30 operational taxonomic units (OTUs), according to the methods described by Nagashima et al. [11]. The OTUs were quantified as the percentage values of an individual OTU per total OTU area, expressed as peak percent area under the curve (%AUC). Cluster analyses were performed using the software GeneMaths (Applied Maths, Belgium), based on the *Bsl*I T-RFLP patterns. The Pearson similarity coefficient analysis and unweighted pair-group method with arithmetic means were used to establish the type of dendrogram.

### Statistical analysis

The patients’ characteristics and nutrient intake profiles were compared between the placebo and 2 TGD groups, using the chi-square test or 2-way ANOVA and Bonferroni post hoc test. The relative abundances of specific bacterial groups were reflected through their T-RF peak areas, and the percentage values were compared between the placebo and 2 TGD groups using the 2-way ANOVA and Bonferroni post hoc test. A p < 0.05 was considered statistically significant.

## Results

**Figure 1:**
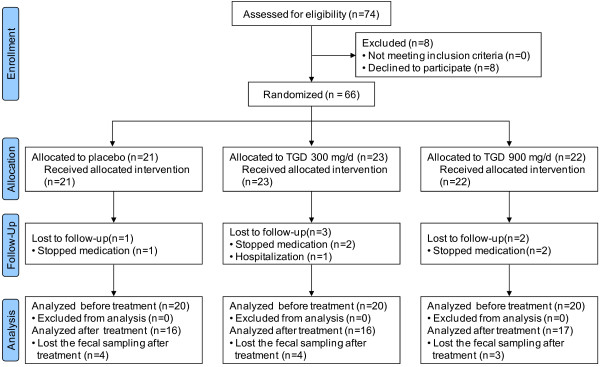
Flow diagram of patients included.

In the previous study, between December 2007 and Marchi 2009, a total of 74 T2DM patients from our outpatient clinic were recruited and 66 (38 men, 28 women) were randomized into the following groups: 21, placebo group; 23, TGD300 group to receive 300 mg of TGD a day; and 22, TGD900 group to receive 900 mg of TGD a day. Six patients did not complete the study, with 5 discontinuing the treatment and 1 was hospitalized due to another disease and left the study. In randomized 66 patients, 60 patients (35 men and 25 women, 20; placebo groups 20; TGD300 group, 20; TGD900 group) whose fecal samples were collected before the treatment were included in the evaluation before treatment. As 11 patients were lost the fecal sampling after treatment, 49 patients (16; placebo group, 16; TGD300 group, 17; TGD900 group) were included in the evaluation after treatment (Figure 1).

The baseline clinical characteristics and biochemical values of the 3 groups had a similar distribution with regard to age, sex, body mass index (BMI), medical history, and biochemical data, including HbA1c, fasting blood glucose, and insulin levels. No significant differences were found between the 3 groups for any of these variables and the nutrient intake profile (Table 1).

**Table 1:** Patients’ characteristics and nutrient intake profiles

	**Placebo (n = 20)**	**TGD 300 mg/day (n = 20)**	**TGD 900 mg/day (n = 20)**	**p value**
Characteristic				
Sex (male/female)	9/11	14/6	12/8	0.272
Age (year)	61.8 ± 8.1	62.4 ± 10.3	62.7 ± 8.3	0.928
Body mass index (kg/m^2^)	22.8 ± 4.2	25.1 ± 4.8	23.2 ± 3.1	0.187
HbA1c (%)	6.9 ± 0.7	6.7 ± 0.5	6.7 ± 0.4	0.319
Fasting blood glucose (mg/dL)	141.4 ± 35.3	143.8 ± 20.1	148.8 ± 23.7	0.677
Insulin (mU/mL)	9.2 ± 8.1	13.7 ± 17.5	13.2 ± 21.9	0.651
Nutrients				
Energy (Kcal)	1565 ± 320	1768 ± 417	1671 ± 321	0.203
Protein (g)	51.3 ± 11.1	56.9 ± 11.7	52.7 ± 8.4	0.203
Fat (g)	39.8 ± 7.3	45.0 ± 10.3	39.3 ± 8.2	0.069
Cholesterol (mg)	254 ± 80	234 ± 53	235 ± 64	0.548
Ongoing diabetes therapies, n (%)				
Insulin injection	3 (15)	2 (10)	6 (30)	0.235
Metoformin	5 (25)	7 (35)	5 (25)	0.720
Insulin secretagogue	13 (65)	12 (60)	9 (45)	0.414
α-Glicosidase inhibitor	8 (40)	10 (50)	10 (50)	0.765
PPAR-γ antagonist	5 (25)	5 (25)	5 (25)	1.000
≥2 diabetes drugs	11 (55)	11 (55)	9 (45)	0.766
Lifestyle modification only	2 (4)	1 (5)	2 (4)	0.804

TGD, transglucosidase; HbA1c, glycolated hemoglobin.

**Figure 2:**
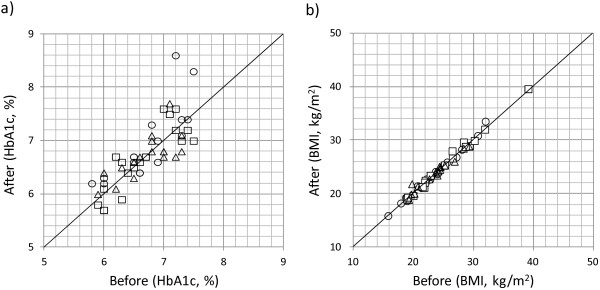
**The HbA1c levels and BMIs before and after the treatment.** Open circle, placebo; open square, TGD300; open triangle, TGD900.

The HbA1c level and BMI before and after the treatment are shown in Figure 2. In the placebo group, HbA1c levels were increased in 11 patients (69%) after the treatment; levels were higher but not significant than those in the TGD-treated group (14 [39%]; p = 0.07). In the placebo group, the BMI had increased in 9 patients (56%) after the treatment; the BMIs were higher than that in the TGD-treated group (14 [39%]).

**Figure 3:**
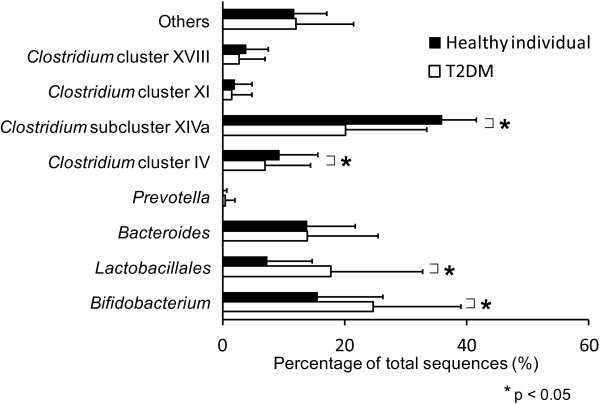
**Gut microbes in the healthy individuals and T2DM patients.** Significant decreases in *Clostridium* cluster IV and *Clostridium* subcluster XIVa components were observed in the T2DM patients compared with the healthy individuals. In contrast, the *Lactobacillales* and *Bifidobacterium* populations significantly increased in the T2DM patients.

A total of 10 healthy individuals (7 men and 3 women) were recruited for comparison of their fecal microbiota with those of the T2DM patients by dendrogram. The %AUC was associated with the predominance of bacterial species that compose each peak. Significant decreases in *Clostridium* cluster IV (OTUs of 168, 369, and 749 bp) and *Clostridium* subcluster XIVa components (OTUs of 106, 494, 505, 517, 754, 955, and 990 bp) were observed in the T2DM patients compared with the healthy individuals. In contrast, the *Lactobacillales* (OTUs of 332, 520, and 657 bp) and *Bifidobacterium* populations (OTUs of 124 bp) significantly increased in the T2DM patients (Figure 3).

**Figure 4:**
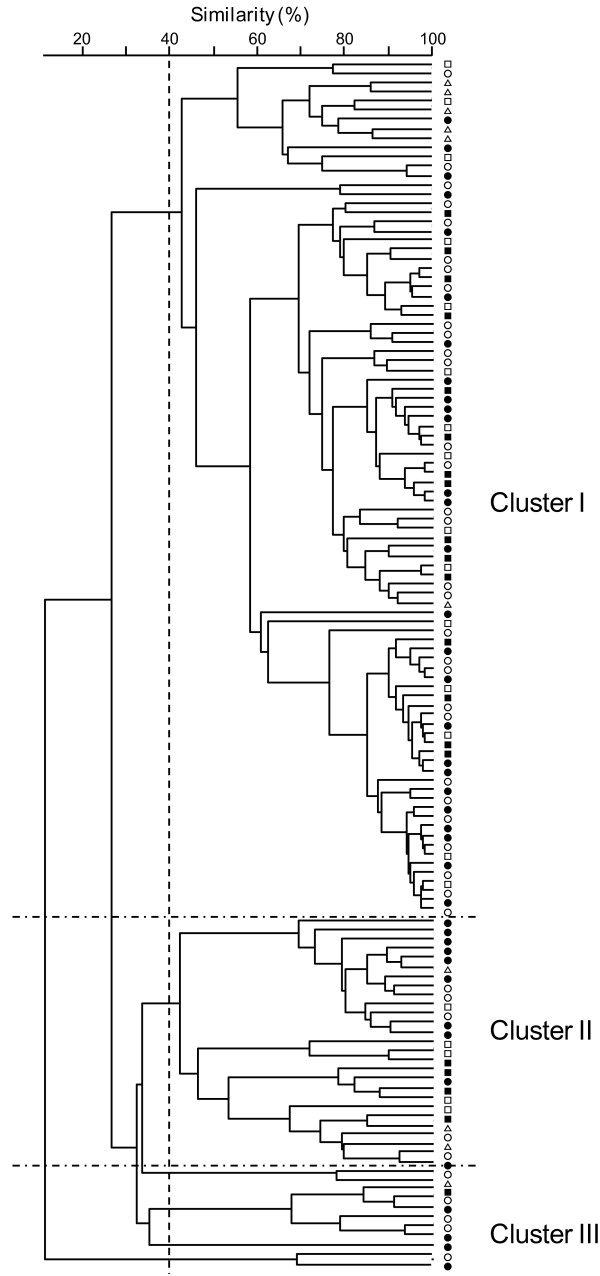
**Dendrogram of the fecal bacteria structure in the T2DM patients and healthy individuals.** T-RFLP patterns by *Bsl*I digestions were analyzed using the software GeneMaths (Applied Maths, Belgium), and the Pearson similarity coefficient analysis and unweighted pair-group method with arithmetic means were used to establish the type of dendrogram. Open triangle, healthy volunteers; open square, T2DM patients before the placebo treatment; closed square, T2DM patients after the placebo treatment; open circle, T2DM patients before the treatment; closed circle, T2DM patients after the treatment.

The fecal bacterial communities were analyzed using a dendrogram. Setting the similarity cutoff at 40% generated 4 major clusters. Most of the healthy individuals (6/10) and T2DM patients (45/60) were classified into cluster I, indicating no significant difference in fecal bacterial communities between the healthy individuals and T2DM patients. In the placebo and TGD groups, the bacterial communities were generally similar before and after the treatment. These results suggest that bacterial lineages were constant within each individual (Figure 4).

**Figure 5:**
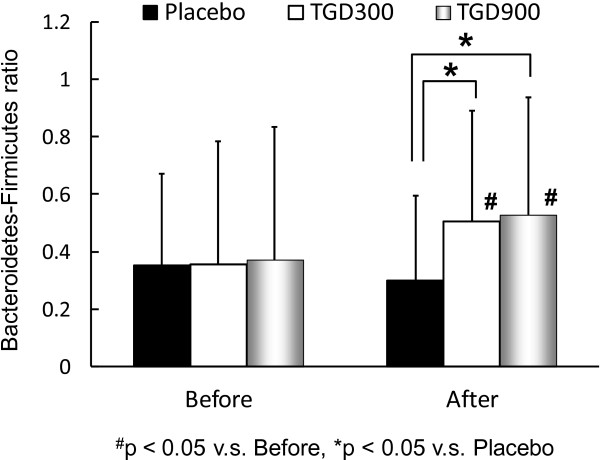
**Bacteroidete*s*-to-Firmicutes ratio before and after the TGD treatment in the T2DM patients.** After the TGD therapy, the Bacteroidetes-to-Firmicutes ratio in both TGD groups significantly increased compared with that before the TGD treatment and was also significantly higher than that in the placebo group.

To investigate the effects of TGD on fecal bacterial communities, the Bacteroidetes-to-Firmicutes ratios in the placebo, TGD300, and TGD900 groups were analyzed (Figure 5). Before the TGD therapy, no significant difference was observed in the Bacteroidetes -to-Firmicutes ratios between the 3 groups. However, the mean size of the Firmicutes populations (49.0%) in the T2DM patients was significantly smaller than that in the healthy patients (58.6%; p < 0.05). After 12 weeks of TGD therapy, the Bacteroidetes-to-Firmicutes ratios in both TGD groups significantly increased compared with that before the TGD treatment and were also significantly higher than that in the placebo group, indicating improvement of gut microbiota. No significant change in Bacteroidetes-to-Firmicutes ratio was observed in the placebo group before and after the treatment. In the group with an increase in Bacteroidetes-to-Firmicutes ratio, 50% (12/24) and 48% (11/23) of the patients had decreased HbA1c levels and BMIs, respectively, after the TGD treatment; however, in the group without an increased Bacteroidetes-to-Firmicutes ratio, only 22% (2/9) and 33% (3/9) of the patients had decreased HbA1c levels and BMIs, respectively, after the TGD treatment.

No serious adverse events were attributable to TGD administration.

## Discussion

T2DM is a metabolic disease primarily caused by obesity-linked insulin resistance. Recent studies have shown a relationship between intestinal microbiota composition and metabolic diseases such as obesity and diabetes. Two groups of beneficial bacteria, Bacteroidetes and Firmicutes, are dominant in the human gut. Ley et al. [6] reported that a relative proportion of Bacteroidetes was decreased in obese people in comparison with lean people and that this proportion increased with weight loss on a low-energy diet. They also demonstrated that obese (*ob*/*ob*) mice had 50% fewer Bacteroidetes and correspondingly more Firmicutes than their lean (+/+) siblings [5]. Backhed et al. [4] transplanted gut microbiota from healthy mice into germ-free recipients, which demonstrated an increase in body fat without any increase in food consumption, raising the possibility that the microbial community composition in the gut affects the amount of energy extract from the diet [4]. These results further indicate that obesity has a microbial component, which might have potential therapeutic implications.

T2DM is a complex disorder influenced by genetic and environmental components. A genome-wide association study is useful for parsing the underlying genetic contributors to T2DM, focusing on identifying genetic components in an organism’s genome. Recently, the risk factors for T2DM was observed to involve other genomes; hence, a metagenome-wide association study should also be performed to analyze intestinal microbiota content [12]. Larsen et al. [12] indicated that the proportion of Firmicutes was significantly reduced in T2DM patients. Schwiertz and colleagues [13] also obtained lower Firmicutes-to-Bacteroidetes ratios in overweight human adults than in lean controls, consistent with our results that smaller Firmicutes populations were observed in the T2DM patients than in the healthy patients. Several studies on mice and human models provided evidence, albeit controversial, that increase in body weight is associated with a large proportion of Firmicutes and relatively small proportion of Bacteroidetes [4,5,7]. In relation to T2DM, the present study demonstrated an increase in *Bifidobacterium* population in the T2DM patients. On the contrary, Wu et al. [14] observed a decrease in *Bifidobacterium* population by PCR-denaturing gradient gel electrophoresis analysis. This discrepancy in results may be due to the difference in methodology between our study and that of Wu et al.

The results of the present trial suggest that the TGD administration improved the body weights and blood glucose levels of the T2DM patients, probably owing to the TGD-induced production of oligosaccharides in the alimentary tract. Dietary fibers of natural and synthetic origins have gained increasing attention because of their beneficial effects of lowering blood glucose and lipid levels and protecting against heart disease [15-17]. However, some reports indicated that dietary fibers have no effect on blood glucose and lipid concentrations [18]. This discrepancy in findings may be due to the reductive effect of oligosaccharide on postprandial hyperglycemia, which is dependent on the composition of the diet [16]. In the present study, because all the diabetic patients received nourishment guidance at the beginning of the treatment, no significant difference in their eating habits was observed, which was confirmed by the questionnaire survey. Using rat gastrointestinal and gastric ligation models, we previously demonstrated that TGD can convert carbohydrates to oligosaccharides [8]. Products such as panose and isomaltooligosaccharide, which are predominantly utilized by bifidobacteria [19], are indigestible oligosaccharides that reach the cecum. We have previously proposed that the consumption of TGD can reduce the total amount of orally ingested calories via the consequent transformation of digestible substrate to indigestible fiber in the alimentary tract [2]. The reduction in total calorie intake and the effect of the oligosaccharides generated by TGD can explain the reduction in blood glucose and lipid concentrations observed in this study. Another mechanism to explain the outcome of this trial may be the improvement in gut microbiota composition, as evident by the increase in the Bacteroidetes-to-Firmicutes ratio. Our study supports the finding of Ley et al. that Bacteroidetes abundance was increased according to the decrease in the body weights of obese individuals by dietary treatment [6].

In summary, we indicated that T2DM in humans is associated with compositional changes in intestinal microbiota, and TGD treatment improved metabolic condition of T2DM and fecal microbiota. These evidence suggests that there is the link between metabolic disease and bacterial population in the gut.

## Conclusions

Therefore, based on the results of the present trial, we suggest that the TGD treatment decreased the blood glucose levels and prevented body weight gain in the T2DM patients by inducing the production of oligosaccharides in the alimentary tract and modulating gut microbiota com-position.

## Competing interests

The authors declare that they have no competing interests.

## Authors’ contributions

ON, FY, MM, and IA coordinated and performed the collection of all the human materials; GC analyzed the results of the data-based short food frequency questionnaire survey; KK and JT edited the manuscript; and SM designed the study and wrote the manuscript. All authors read and approved the final manuscript.

## Pre-publication history

The pre-publication history for this paper can be accessed here:

http://www.biomedcentral.com/1471-230X/13/81/prepub
